# Declining mortality following acute myocardial infarction in the Department of Veterans Affairs Health Care System

**DOI:** 10.1186/1471-2261-9-44

**Published:** 2009-08-31

**Authors:** Stephan D Fihn, Mary Vaughan-Sarrazin, Elliott Lowy, Ioana Popescu, Charles Maynard, Gary E Rosenthal, Anne E Sales, John Rumsfeld, Sandy Piñeros, Mary B McDonell, Christian D Helfrich, Roxane Rusch, Robert Jesse, Peter Almenoff, Barbara Fleming, Michael Kussman

**Affiliations:** 1 VA Puget Sound Health Care System, Seattle, WA, USA; 2 Department of Medicine, University of Washington, Seattle, WA, USA; 3 Department of Health Services, University of Washington, Seattle, WA, USA; 4 VA Medical Center, Iowa City and Department of Medicine, University of Iowa, Iowa City, IA, USA; 5 University of Alberta, Edmonton, Alberta, Canada; 6 VA Medical Center, Denver, CO, USA; and Department of Medicine, University of Colorado, Denver, CO, USA; 7 Department of Veterans Affairs, Washington DC, USA

## Abstract

**Background:**

Mortality from acute myocardial infarction (AMI) is declining worldwide. We sought to determine if mortality in the Veterans Health Administration (VHA) has also been declining.

**Methods:**

We calculated 30-day mortality rates between 2004 and 2006 using data from the VHA External Peer Review Program (EPRP), which entails detailed abstraction of records of all patients with AMI. To compare trends within VHA with other systems of care, we estimated relative mortality rates between 2000 and 2005 for all males 65 years and older with a primary diagnosis of AMI using administrative data from the VHA Patient Treatment File and the Medicare Provider Analysis and Review (MedPAR) files.

**Results:**

Using EPRP data on 11,609 patients, we observed a statistically significant decline in adjusted 30-day mortality following AMI in VHA from 16.3% in 2004 to 13.9% in 2006, a relative decrease of 15% and a decrease in the odds of dying of 10% per year (p = .011). Similar declines were found for in-hospital and 90-day mortality.

Based on administrative data on 27,494 VHA patients age 65 years and older and 789,400 Medicare patients, 30-day mortality following AMI declined from 16.0% during 2000-2001 to 15.7% during 2004-June 2005 in VHA and from 16.7% to 15.5% in private sector hospitals. After adjusting for patient characteristics and hospital effects, the overall relative odds of death were similar for VHA and Medicare (odds ratio 1.02, 95% C.I. 0.96-1.08).

**Conclusion:**

Mortality following AMI within VHA has declined significantly since 2003 at a rate that parallels that in Medicare-funded hospitals.

## Background

The Department of Veterans Affairs (VA) operates the largest integrated health care system in the U.S. including 154 medical centers in all 50 states and Puerto Rico. Over the past 12 years the Veterans Health Administration (VHA) has undergone a remarkable transformation accompanied by remarkable improvements in quality. [1] In a study of nearly 600 patients receiving care from VHA in 12 communities between 1997 and 2000, their health care was generally of higher quality on 294 measures than that received by nearly 1000 patients enrolled in commercial managed care plans in the same communities.[2] More recently, VHA's performance on a wide range of measures that are monitored for patients enrolled in Medicare, Medicaid, and commercial managed care plans (Health Employer Data Set - HEDIS) outpaced that of health care plans in these settings. [3] On most of the ORYX measures publicly reported by The Joint Commission, VHA's most recent average national scores equaled or exceeded mean scores for non-VA facilities.[4] These achievements reflected by performance measures are paralleled by data indicating that adjusted mortality was lower over a four-year period for patients receiving care in VHA compared with those enrolled in the Medicare Advantage program. [5] These efforts have led to VHA's recognition as one of the highest quality health care systems in the nation [6-9], although others have questioned their validity. [10]

In the area of cardiac care, studies performed a decade ago found that mortality following acute myocardial infarction (AMI) among patients treated in VHA facilities was similar to that of patients whose care was funded by Medicare in non-VA hospitals. [11,12] Subsequently, in an external study commissioned by VA that used administrative data from patients hospitalized with AMI between 1996 and 1999, Landrum and colleagues found that survival was significantly lower at one year than for patients whose care was funded by Medicare. [13] Despite concerns about that study's methodology, VA used the study as an impetus for an ambitious 10-point cardiac care initiative intended to improve acute cardiac care.[14]

A subsequent study, however, showed that the higher mortality within VHA reported by Landrum, et al was likely an artifact related to a much higher proportion of patients who suffered an AMI after being admitted to the hospital for another medical problem in the VA system than in the Medicare cohorts. [15] Given these controversies, we sought to reevaluate trends in mortality since the VA cardiac care initiative was undertaken. Moreover, because mortality related to cardiac disease has been declining worldwide, we also were interested in comparing trends within VHA with the Medicare population as an indication of secular trends. [16]

## Methods

We undertook two separate sets of analyses: one to calculate annual mortality rates in VHA following AMI and a second to compare mortality rates among patients treated for AMI in VHA hospitals and those in hospitals funded by Medicare (Table 1).

**Table 1:** Description of studies performed

**Study and Comparisons**		
		
	**Trends in AMI mortality within VHA**	**Trends in AMI mortality within VHA and Medicare**
**Time Period**	1/1/2004 - 9/30/2006	1/1/2000 - 6/30/2005

**Study sample**	All patients ≥65 years old with ICD-9-CM codes 410.xx	All patients ≥65 years old with ICD-9-CM codes 410 except 410.x2

**Sample size**	11,609 patients	27,494 VHA patients and 789,400 Medicare patients 789,400 Medicare patients

**Data Sources**		

**Identification of patients**	Abstracted record of all patients with AMI (External Peer Review Program)	VA Patient Treatment File (PTF); Medicare Provider Analysis and Review (MedPAR) File Provider Analysis and Review (MedPAR) File

**Mortality**	VA Vital Status File	MedPAR; VA Vital Status File

### Trends in Mortality in VHA

To examine trends in mortality following AMI within VHA we used information from the VHA External Peer Review Program (EPRP), which is a quality monitoring and improvement system for a variety of medical conditions and procedures, including AMI. All patients who were age 65 years and older and who had been assigned *International Classification of Diseases, 9th Revision: Clinical Modification (ICD-9-CM)*, diagnostic codes 410.xx at 145 VHA hospitals during the period January 1, 2004 through September 30, 2006 were identified from administrative data housed at the Austin Automation Center, Austin, TX. Trained abstractors under contract to the EPRP program reviewed these records to validate the coded diagnoses and collect extensive clinical information. We used information from EPRP and VA workload files to describe demographic and personal characteristics (e.g., age, sex, ethnicity, body mass index and distance from the veteran's residence to the medical center), receipt of reperfusion therapy, and use of cardiac procedures. [17,18] Ethnicity was classified as African-American, Hispanic, White, other, or unknown. Clinical data, such as medical history, cardiac medications administered, time from symptom onset to hospital admission, initial heart rate, systolic and diastolic blood pressures, and initial symptoms, were obtained from the medical record. Patients included in the analysis were males age 65 or older who presented to a VHA facility with an AMI. Patients who developed evidence of AMI during hospitalization were excluded.

The initial electrocardiographic diagnosis of ST-segment elevation AMI included patients with ST-segment elevation of 1 mV or higher in 2 or more contiguous leads and/or left bundle branch block. The remainder of patients had non-ST-segment elevation AMI. Both the initial and highest troponin values were recorded; positive tests were determined according to criteria for the particular assay used. In both groups, more than 99% of the AMIs were confirmed by troponin level elevations.

#### Ascertainment of Vital Status

The primary endpoint was all-cause mortality within 30 days of admission. Secondary endpoints were in-hospital, 90-day, and 1-year mortality. Vital status was ascertained using the VA vital status file, which melds four separate sources: VA Beneficiary Identification and Record Locator System (BIRLS); VA Patient Treatment File (PTF); Social Security Administration death master file; and Medicare vital status file. The VA vital status file has been shown to have a sensitivity of 98.3% and specificity was 99.8% in comparison with the National Death Index. [19]

#### Analysis

We applied a logistic regression model for 30-day mortality that was developed and validated expressly for VA patients using earlier versions of EPRP data. [15] The model includes history of cancer, history of dementia, history of heart failure, history of stroke within 5 years, history of lipid disorder or prescriptions for lipid medications, age, elevated initial troponin, creatinine, heart rate, systolic blood pressure, chest and/or shoulder pain, and presentation at night. Analyses were run with and without a cluster correction for medical center.

### Comparisons of Trends in VHA with Medicare

We used comparable administrative data to estimate the relative mortality rates among patients admitted with AMI to VHA medical centers versus patients whose care was covered by Medicare at non-VA hospitals. Data were derived from two primary sources: the Medicare Provider Analysis and Review (MedPAR) data files; and the VA Patient Treatment File. The MedPAR files contain administrative data on all Medicare fee-for-service hospitalizations, including: demographic information; admission source (e.g., transfer from another hospital, emergency room); ZIP code; primary and secondary diagnoses and procedures, as defined by ICD-9-CM codes; hospital admission and discharge dates; disposition at the time of hospital discharge; and a six-digit unique hospital identifier. The MedPAR files are matched quarterly to the Medicare Enrollment database to incorporate dates of death after hospital discharge. The Patient Treatment File contains data on all hospitalizations in VA medical centers nationally and includes similar data elements as the MedPAR files. The US Census 2000 Summary File was the source for ZIP code-level socioeconomic measures (e.g., median household income). In order to identify non-VA hospitals in the same geographic markets as VA hospitals, we used the Dartmouth Atlas to map each VA and non-VA hospital to one of 306 distinct hospital referral regions (i.e., health care markets) for tertiary health services. [20,21]

#### Patients

VA patients were comprised of all males 65 years and older with a primary diagnosis of AMI (ICD-9-CM code 410, excluding 410.x2) who were admitted between January 1, 2000 and June 30, 2005 to 145 VA hospitals (n = 33,632). Medicare patients were similarly defined and included 4,580 non-VA hospitals (n = 955,780), with 2,876 hospitals located in 112 geographic markets with a VA hospital. We limited the analysis to males, given the preponderance of males (98.5%) in the VA sample. We further excluded 146,953 (14.8%) patients who were admitted as transfers from other acute care hospitals and 25,565 (2.6%) patients who could not be matched to ZIP code-level socioeconomic measures.

The analysis incorporated a variety of characteristics including demographic variables such as age, ethnic group (categorized as White, Black, Hispanic, other, or missing), median household income (based on residential ZIP code-level data), admission from a skilled nursing facility, and distance from the patient's residence to the admitting hospital (based on distance between ZIP code centroids). Clinical variables included coexisting conditions listed in Table 4 (defined by ICD-9-CM secondary diagnosis codes using previously defined algorithms for administrative data developed by Elixhauser, et al [22] and Hannan, et al [23]; and type of AMI (categorized as anterior or lateral, inferior or posterior, subendocardial, or other unspecified location (defined by the fourth digit of the primary ICD-9-CM code)).

#### Ascertainment of Vital Status

Mortality after 30 days among patients treated within VHA was established using the VA Vital Status File, while mortality of Medicare patients was determined using the date of death recorded in Medicare enrollment files.

#### Analysis

Characteristics of patients that were related on a bivariate basis (p < .01) to each of the mortality endpoints were entered into stepwise logistic regression models. We eliminated predictors that were not significant (p ≥ .01) or which exhibited effects that were not parsimonious with expected clinical effects. Remaining predictors were entered into subsequent logistic regression models that also included discharge year (categorized as 2000-2001, 2002-2003, and 2004-June 2005) and an indicator variable for admission to a VA hospital. Models included random intercepts for hospitals. Thus, the exponentiated value of the VA hospital indicator represents the odds of death in the "average" VA hospital relative to the "average" private sector hospital. Models were also generated separately for patients discharged during 2000-2001, 2002-2003, and 2004-June 2005. Finally, analyses were conducted for all patients nationwide and for patients who were admitted to the 112 hospital referral regions that included VA hospitals.

SPSS version 13.0, SAS 9.0, and Stata version 9.0 statistical software (SPSS Inc, Chicago, IL, and StataCorp, College Station, TX, respectively) were used for all analyses reported. This study was approved by the Institutional Review Boards of the University of Washington, Seattle, and University of Iowa, Iowa City, and waivers of informed consent were granted.

## Results

### Trends in Mortality in VHA

Based upon VA EPRP data, there were a total of 11,609 patients with AMI available for analysis including 4411 in 2004, 4412 in 2005 and 2786 in 2006. The mean age was 76.9 years (Table 2). Over half the patients were White, but sizable proportions were African-American (9.4%) or Hispanic (6.9%) while ethnic classification was unknown in 25.3%. There was a high prevalence of preexisting cardiac disease as evidenced by the fact that one-quarter of patients had a prior history of AMI, one-third carried a diagnosis of heart failure, and nearly one-quarter had undergone coronary artery bypass surgery. Most patients had multiple cardiac risk factors including lipid abnormalities (64.3%), smoking (13.5%) and diabetes (63.4%). Many patients were receiving cardioprotective medications such as aspirin, β-blockers, angiotensin-converting enzyme inhibitors or lipid-lowering drugs at the time of admission.

**Table 2:** Characteristics of Patients with AMI treated in VHA

Mean age, mean ± SD, y	76.9 ± 6.9
Age, %	
65-69 y	16.9
70-79 y	46.1
80-89 y	34.1
>90 y	2.9
Ethnicity, %	
Hispanic	6.9
African American	9.4
White	57.8
Other	0.6
Unknown	25.3
Distance from home to hospital in miles, mean ± SD	39 ± 117
Geographic region of medical center, %	
New England	3.2
Mid-Atlantic	10.0
Great Lakes	10.6
North Plants	8.7
South Atlantic (including Puerto Rico)	28.3
Mississippi	7.7
South Plains	11.5
Great Basin	9.5
Pacific	10.6
Medical history, %	
Myocardial infarction	26.5
Lipid disorder	64.3
Coronary angioplasty/PCI w/in 6 mos	3.5
Coronary artery bypass surgery	23.8
Heart failure	37.7
Diabetes mellitus	63.4
Renal disease	18.6
Cerebrovascular disease	9.6
Chronic obstructive pulmonary disease	21.7
Dementia	16.3
Cancer	11.5
Current smoking	13.5
Medications at the time of admission for AMI, %	
Aspirin	49.8
Beta-blocker	57.3
Angiotensin-converting enzyme inhibitor	48.4
Lipid-lowering drug	54.9
Insulin	15.6

2004-2006 (n = 11,609)

**Figure 1:**
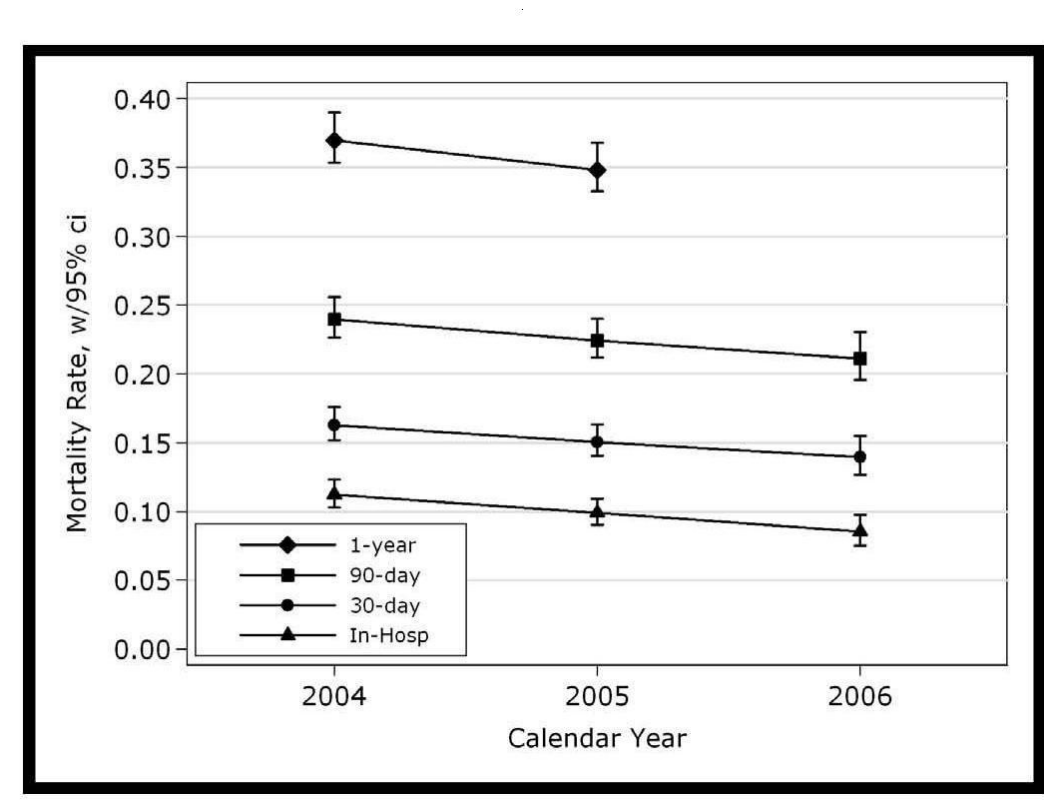
**Adjusted mortality with 95% confidence intervals following AMI in VHA 2004 through 2006 (N = 11,609)**.

Using detailed clinical data to adjust for demographic and clinical factors, we observed a statistically significant decline in 30-day mortality following AMI from 16.3% in 2004 to 13.9% in 2006, a relative decrease of 15% and a decrease in the odds of dying of 10% per year (p = .011, Table 3 and Figure 1). Similar declines were found for in-hospital and 90-day mortality.

**Table 3:** Adjusted mortality following AMI in VHA

		**2004**			**2005**			**2006**		**Overall**
	**Obs**	**Pre**	**Adj**	**Obs**	**Pre**	**Adj**	**Obs**	**Pre**	**Adj**	**Obs**
**In-hospital**	0.110	0.099	0.112	0.102	0.103	0.099	0.084	0.099	0.085	0.101
**30-day**	0.160	0.150	0.163	0.153	0.155	0.151	0.138	0.151	0.139	0.152
**90-day**	0.237	0.224	0.240	0.228	0.230	0.225	0.209	0.225	0.211	0.227
**One-year**	0.365	0.354	0.370	0.352	0.362	0.349	NA	NA	NA	0.359

*Adjustments made for age, heart rate, systolic pressure, persistent chest pain, hyperlipidemia, serum creatinine, elevation of initial troponin determination, medical history (heart failure, dementia, cancer or stroke within the past 5 years), and presentation at night. Adjusted mortality is Observed/Predicted * overall mortality for each measure. NA = not available, Obs = observed, Pre = predicted.

2004 through 2006* (N = 11,609)

### Comparisons of Trends in VHA with Medicare

Declines in mortality following AMI within VHA hospitals were compared with those experienced in private sector hospitals using administrative data from VHA and CMS. The final sample included 27,494 patients age 65 years and older admitted to VHA medical centers, and 789,400 Medicare patients admitted to private sector hospitals for AMI during the period 2000 to June 2005, with 500,796 (63.4%) Medicare patients in hospitals in the same geographic region as a VA Hospital.

VHA patients were on average a year and one-half younger than private sector Medicare patients (mean ages 75.7 vs. 77.1, Table 4), were less apt to be White, and had a substantially lower median annual income. Patients admitted to VHA hospitals were more likely to have previous diagnoses of cerebrovascular disease, COPD, diabetes, liver disease, depression, or hypertension while Medicare patients were more likely to have cardiac arrhythmia, kidney or heart failure, or neurologic disorder. AMIs in patients in the Medicare cohort were more likely to be in anterior/lateral or inferior/posterior locations while AMIs in the VA cohort were more likely to classified as subendocardial or "other."

**Table 4:** Characteristics of male VHA and Medicare patients

	**VHA**	**Medicare**
**Total no. of patients**	27,494	789,400
		
**Mean age, yrs (s.d.)**	75.7 (6.4)	77.1 (7.7)
**Age 65-69, %**	20.1	19.7
**Age 70-74, %**	24.6	20.9
**Age75-79, %**	26.7	21.9
**Age 80-84, %**	19.6	18.9
**Age 85-89, %**	7.1	12.2
**Age 90 plus, %**	1.9	6.4
		
**White, %**	69.4	89.9
**Median income (/$10,000) for ZIP code (s.d.)**	37.1 (13.3)	42.5 (15.7)
		
**Distance to admitting facility**	32.2 (55.6)	31.7 (61.5)
		
**Admission from extended care facility, %**	4.0	0.7
**Coexisting Conditions, %**		
**Cardiac Arrhythmia**	22.7	31.2
**Paralysis**	0.7	0.4
**Neurological Disorder**	4.0	5.4
**Coagulation Disorder**	2.4	3.3
**Weight Loss**	1.4	1.1
**Psychosis**	2.1	0.9
**Renal Failure**	8.6	9.2
**Chronic Heart failure**	33.3	39.9
**Cerebrovascular disease**	8.3	6.7
**Chronic obstructive lung disease**	25.7	24.4
**Liver Disease**	0.8	0.5
**Depression**	3.2	2.0
**Diabetes**	37.1	27.3
**Diabetes with complication**	5.4	3.8
**Hypertension**	53.8	43.4
		
**Location of AMI,%**		
**Anterior/Lateral**	8.6	13.9
**Inferior/Posterior**	10.1	16.3
**Subendocardial**	59.1	57.9
**Other Site**	22.1	11.9
		
**Year of Discharge**		
**2000**	17.9	18.8
**2001**	19.1	18.8
**2002**	18.9	18.8
**2003**	17.8	18.3
**2004**	17.3	16.8
**2005 (Through June)**	9.1	8.4

aged 65 years or older admitted with AMI, June 2000 through June 2005*

For the period January 1, 2000 through June 30, 2005, overall unadjusted 30-day mortality was 16.0% in VHA hospitals and 16.2% in private sector hospitals (Table 5). Mortality declined in both cohorts, from 16.7% during 2000-2001 to 15.5% during 2004-June 2005 for private sector hospitals, and from 16.0% to 15.7% during that same period for VHA hospitals. After adjusting for patient characteristics and hospital effects, the overall relative odds of death were not significantly different for VHA or private sector hospitals (odds ratio 1.02, 95% 0.96-1.08, Table 6). Moreover, we observed no differences in analyses conducted separately using patients discharged during 2000-2001, 2002-2003, and 2004-June 2005, or when we restricted analyses to hospitals located within a market that contains a VHA hospital.

**Table 5:** Unadjusted 30-day mortality rates

	**Patients in 145 VHA hospitals**		**Medicare patients in 4580 private sector hospitals**		**Medicare patients in 2876 hospitals in 112 Markets withVHA**	
						
**Discharge Year**	**N**	**Mortality**	**N**	**Mortality**	**N**	**Mortality**
**2000-2001**	10,165	16.0%	297,121	16.7%	179,279 179,279	16.8%
**2002-2003**	10,088	16.3%	292,833	16.2%	175,443	16.3%
**2004-June 2005**	7,241	15.7%	199,446	15.5%	118,580	15.6%
**All years**	27,494	16.0%	789,400	16.2%	473,302	16.3%

Following admission for AMI in VHA and Medicare, 2000-2005

**Table 6:** Adjusted odds of death

**Odds Ratio (95% CI, p-value)**		
		
**Discharge Period**	**Relative to Private Sector Hospitals** **in all markets**	**Relative to Private Sector hospitals in 112 markets with VHA**
**2000-June 2005**	1.02 (0.96-1.08; p = .60)	1.01 (0.95-1.07; p = .71)
**2000-2001**	1.00 (0.92-1.08; p = .91)	1.00 (0.93-1.08; p = .91)
**2002-2003**	1.05 (0.98-1.13; p = .15)	1.05 (0.97-1.12; p = .18)
**2004-June 2005**	1.08 (0.98-1.19; p = .10)	1.07 (0.97-1;18; p = .14)

Within 30 days following AMI in VHA hospitals relative to all private sector hospitals and relative to private sector hospitals in markets with a VHA hospital: January 1, 2000-June 30, 2005.

## Discussion

Using both administrative data and detailed clinical information abstracted from medical records, we found that mortality following AMI among patients admitted to VA medical centers significantly declined between 2004 and 2006. This decline approximates the decrease that has occurred in hospitals outside of the VHA system during the same timeframe. These results are consistent with reports from a decade ago indicating parity in AMI mortality between VHA and Medicare. Our findings appear to lay to rest concerns that were raised more recently that mortality among patients treated for AMI in VHA appeared significantly higher than for patients whose care was funded by Medicare. In that study, which was actually commissioned and funded by VHA, administrative data from 1997 and 1998 were analyzed to evaluate mortality for patients admitted with AMI. The investigators reported that 30-day mortality in the 1997 cohort was 18.4% in VA and 14.9% in Medicare while figures for one-year mortality were 34.5% and 28.0% respectively. The gap was reported to narrow somewhat in 1998 with 30-day mortality estimates of 16.3% and 14.8% for VHA and Medicare, respectively, and one-year estimates of 34.0% and 28.7%.

A subsequent study, however, indicated that findings of high post-AMI mortality in VA patients were biased, because patients who suffered an AMI after being admitted to the hospital for another problem were far more prevalent in the VHA than in Medicare cohorts. Using updated data and methods in previous work, we have found that in-hospital AMI accounts for 11% of all AMIs within the VHA system and the mortality for this problem is 28%, nearly fourfold higher than for patients who are admitted initially for an evolving AMI. [15] When all patients with in-hospital AMI were excluded from consideration, 30-day mortality following AMI was found to be 11.9%, which is lower than observed in the present analysis but includes patients younger than age 65.

Although the decrease in mortality within VA was commensurate with that occurring in the rest of the country, it may, in part, have been related to an aggressive effort undertaken within VHA over the past four years. Following public release of the data by Landrum, et al, VHA embarked upon a system-wide cardiac care initiative that encompassed educational programs for patients and providers, expansion of performance measurement and quality improvement systems, creation of administrative structures to coordinate care for AMI, and enhancement of infrastructure such as cardiac catheterization laboratories.[14] There are, however, other explanations as well. The most likely is that VHA has maintained very high levels of performance with regard to prescribing cardioprotective medications post-AMI. Data from 2006 showed that eligible patients in VHA received aspirin, statins, and β-blockers at discharge at least 95% of the time, and that 87% of those with left-ventricular ejection fractions below 40% received angiotensin converting enzyme inhibitors. These figures exceeded performance of all hospitals reporting to the Joint Commission.[4] Moreover, investigators have found that VA patients tend to be substantially more adherent to these medication regimens over time, presumably because their cost of medications is so much lower than that of most patients enrolled in Medicare.[24]

There have been concerns that patients in VA were less likely to undergo indicated cardiac interventions after AMI. [25] In 2005, 39% of eligible patients with AMI received thrombolytic therapy within 30 minutes (39%), which is comparable to all hospitals reporting to the Joint Commission. However, VA currently reports a lower proportion of patients who undergo percutaneous coronary interventions within 90 minutes when indicated (48% vs. 78%). Although this latter difference is clinically important, it would be likely to translate into only very small differences in overall survival following AMI. Less than one-quarter of patients admitted to VA hospitals with AMI have ST-segment elevation, and of these, only a relatively small fraction present early enough to be eligible for emergent PCI. Compared with noninvasive treatment, emergent PCI improves short-term mortality by approximately 2% in absolute terms. [26,27] Moreover, Ford, et al estimate that pharmacologic interventions and rehabilitation during and after hospitalization are responsible for 93% of the reduction in mortality following AMI while only 7% is attributable to revascularization. [28] More aggressive early treatment of AMI thus is likely responsible for only a relatively small proportion of the observed declines in mortality in either VA or Medicare patients.

It could be argued that some of the observed decline in mortality is simply due to increasing use of "super-sensitive" troponin assays. These assays could be responsible for increasing detection of patients with minimal myocardial damage whose prognosis is good. However, the data are inconsistent with this hypothesis. Existing evidence suggests that elevated troponin, even in the absence of classic indicators of AMI, confers a substantially increased risk of short-term mortality so that including these patients would not necessarily bias mortality downward. [29] Furthermore, increasing the detection of NSTEMI AMIs would be expected to increase the proportion of AMIs that are NSTEMI and to increase the overall number of AMIs. In the data reported here, the proportion of AMIs that were NSTEMI increased only slightly from 73% to 76% while the absolute number of AMIs actually declined.

To determine whether the observed declines could be explained by increasing inclusion of patients with small, low-risk infarcts diagnosed by slight elevations of serum troponin, we performed a regression of troponin level on discharge date. Approximately 200 assay type/hospital groups were excluded because there were less than three measurements, leaving approximately 9000 of 11,000 troponin values for analysis. Although we obtained a regression coefficient of about -0.07 per year (p < 0.0001) for initial and peak troponins, the R^2 ^was quite small (0.003). And finally, we performed an informal analysis of 9000 troponin values in an attempt to ascertain whether there was a trend toward lower values over time. Although the analysis was complicated by the fact that patients varied widely with respect to the number of tests performed and their timing, date of discharge accounted for only 0.3% of the variance in mean initial and peak troponin levels.

This study has several strengths that include eligibility of all patients diagnosed with AMI in both VA and Medicare populations over a period of several years and careful adjustment of mortality within VHA using two completely different approaches. In addition, we ascertained mortality using methods that have been shown to be highly reliable and valid. There are, however, significant potential limitations that deserve comment. First, the EPRP data used to assess mortality following AMI in VHA were available only for calendar years 2004 through 2006. Second, we were able to compare mortality in VHA and Medicare using only administrative data because CMS does not collect detailed clinical data on all patients with AMI. Although there may have been unrecognized differences in the way these data were collected, we believe that measurement of vital status is consistent for the two populations. Third, there is ample evidence that reliance solely on administrative data may fail to adequately adjust for differences among patients treated in different systems of care. [30] We think this is unlikely to have seriously affected our analyses given that both the crude and fully adjusted estimates of mortality were similar in the VA and Medicare groups. Fourth, because the public release of Medicare data is delayed, we were only able to make these comparisons through 2005. Finally and most importantly, the inherent differences in the VA EPRP data and the VHA and CMS administrative data sources led to slightly different denominators and estimates of outcome. The lower number of patients with AMI in the EPRP analysis is related to the fact that in a substantial minority of patients who have a discharge diagnosis of AMI, careful review of the medical record does not substantiate that diagnosis. The differences in adjusted outcomes result, in part, from the ability to adjust more completely for the considerable burden of coexisting, chronic illness among VA patients.

## Conclusion

In summary, we found that mortality following AMI has declined over the past four years in VA and that this decline has been commensurate with that occurring in the Medicare population.

## Competing interests

The authors declare that they have no competing interests.

## Authors' contributions

The authors declare that they have no competing interests and all authors have read and approved the final manuscript. SDF conceived of the study, participated in design and analysis and drafted the manuscript. MVS and EL participated in the design of the study, data collection, performed statistical analysis, and helped draft the manuscript. IP, CM, GR, AS and JR were involved in study conception, design, and analysis and helped draft the manuscript. SP, MM, CH, and RR participated in study coordination, data collection and helped draft the manuscript. RJ, PA, BF and MK participated in study design, and helped draft the manuscript.

## Pre-publication history

The pre-publication history for this paper can be accessed here:


